# Adult‐Onset Bartter Syndrome Presenting as Refractory Hypokalemia and Metabolic Alkalosis: A Case Report

**DOI:** 10.1002/ccr3.72541

**Published:** 2026-04-14

**Authors:** Muhammad Asif, Ayesha Sanam, Muhammad Ilyas, Sawera Tahir, Tahir Hanif, Muddassir Khalid, Iqra Sarwar, Fred Segawa

**Affiliations:** ^1^ Registrar Medicine Benazir Bhutto Hospital Rawalpindi Pakistan; ^2^ Department of Biochemistry Army Medical College NUMS Rawalpindi Pakistan; ^3^ Internal Medicine Trainee Daisy Hill Hospital UK; ^4^ Clinical Fellow Ysbyty Gwynedd Bangor Wales UK; ^5^ Department of Medicine CMH Lahore Pakistan; ^6^ Department of Medicine Nishtar Medical University Multan Pakistan; ^7^ Department of Medicine Makerere University College of Health Sciences Kampala Central Region Uganda

**Keywords:** autosomal recessive salt‐wasting tubulopathy, bartter syndrome, case report, hypokalemia, metabolic alkalosis

## Abstract

Bartter syndrome (BS) is a rare autosomal recessive salt‐wasting tubulopathy characterized by hypokalemic metabolic alkalosis, hyperreninemia, and hyperaldosteronism without hypertension. It usually presents in childhood; however, adult‐onset cases are infrequent and often misdiagnosed. We report a young adult male from Pakistan with congenital deaf‐mutism who presented with vomiting, irritability, and altered sensorium. Laboratory findings revealed persistent hypokalemia, metabolic alkalosis, and acute kidney injury secondary to sepsis. Despite adequate potassium replacement, hypokalemia persisted, prompting evaluation for a renal potassium‐wasting disorder. Further investigations showed elevated urinary sodium, potassium, and chloride with normal magnesium and a normal‐to‐high urinary calcium‐to‐creatinine ratio, consistent with Bartter syndrome. The patient was managed with intravenous fluids, antibiotics, and potassium supplementation, followed by the addition of oral potassium chloride and spironolactone. Clinical and biochemical improvement was noted, with normalization of renal function and serum electrolytes at discharge and on follow‐up. This case underscores the diagnostic importance of considering Bartter syndrome in adults with refractory hypokalemia and metabolic alkalosis, particularly after excluding more common causes such as vomiting or diuretic abuse. The coexistence of chronic hypotension and prior myocardial infarction highlights the potential cardiovascular implications of chronic hypokalemia. Bartter syndrome may present in adulthood with atypical features. Early recognition through targeted biochemical evaluation and timely initiation of potassium‐sparing therapy are crucial for favorable outcomes and prevention of renal and cardiovascular complications, especially in resource‐limited settings lacking genetic diagnostic facilities.

## Key Clinical Message

Bartter syndrome can present in adults with refractory hypokalemia and metabolic alkalosis. A high index of suspicion is essential when hypokalemia persists despite adequate replacement, particularly in normotensive patients. Diagnosis relies on characteristic urinary electrolyte findings, and timely initiation of potassium‐sparing diuretics is crucial for effective management and preventing long‐term complications.

## 1 Introduction

Bartter syndrome (BS) is a rare autosomal recessive salt‐wasting tubulopathy caused by defective ion transport in the thick ascending limb of the loop of Henle, leading to significant renal salt loss [1]. First described by Frederic C. Bartter in 1962, it is characterized by hypokalemic metabolic alkalosis, hyperreninemia, and hyperaldosteronism in the absence of hypertension [2]. BS comprises five genetic subtypes, all involving impaired salt reabsorption in the TAL but differing in their underlying gene mutations and overlapping clinical features [3].

The fundamental defect in Bartter syndrome (BS) is impaired sodium, potassium, and chloride reabsorption in the thick ascending limb (TAL) of the loop of Henle, leading to renal salt wasting, hypokalemia, and metabolic alkalosis [4]. BS results from mutations in genes encoding the Na^+^‐K^+^‐2Cl^−^ cotransporter (SLC12A1), potassium channel (KCNJ1), chloride channels (CLCNKA, CLCNKB), the β‐subunit BSND, or regulatory protein MAGE‐D2. Defective chloride transport at the macula densa disrupts tubuloglomerular feedback, causing persistent stimulation of the renin angiotensin aldosterone system and overproduction of prostaglandin E₂, which together lead to hyperreninemic hyperaldosteronism despite normotension [5].

## 2 Case History

A young adult male, congenitally deaf and mute, was brought to the Medical Unit II, Benazir Bhutto Hospital, Rawalpindi, with a 3‐day history of projectile, yellowish vomiting containing food particles, followed by progressive irritability and altered sensorium. There was no associated history of seizures, headache, trauma, fever, cough, diarrhea, or rash. His attendants reported longstanding polyuria (8–10 times per day), which had gradually decreased over the preceding days, culminating in anuria for 24 h prior to admission. His past history was significant for an inferior wall myocardial infarction two years earlier, managed conservatively, though he had discontinued cardiac medications 10 months prior. There was no history of surgical procedures, blood transfusions, or other chronic illnesses. Family history revealed parental consanguinity, but his siblings were reported to be healthy. He belonged to a lower socioeconomic background, was uneducated, worked as a farmer, and used Nicotiana tabaccum (Naswar) but had no other addictions. On examination, he was a young adult of average build, lying unconscious, with mild icterus and poor oral hygiene. He was persistently hypotensive, with an average systolic blood pressure around 90 mmHg. His peripheral temperature was warm and systemic examination revealed no focal neurological deficits.

### 2.1 Differential Diagnosis

- Surreptitious diuretic use (loop or thiazide)
- Gitelman syndrome
- Chronic vomiting/gastric fluid loss
- Congenital chloride diarrhea.
- Primary hyperaldosteronism (Conn's syndrome)
- Renin‐secreting tumor (reninoma)
- Renovascular hypertension
- Cushing syndrome/cortisol excess
- Liddle syndrome
- Magnesium deficiency–induced renal potassium wasting
- Pseudo‐Bartter states (e.g., cystic fibrosis, laxative abuse, eating disorders)
- Drug‐induced renal salt wasting (aminoglycosides, cisplatin, amphotericin B)
- Other inherited tubulopathies (e.g., EAST/SeSAME syndrome, CaSR gain‐of‐function disorders)

### 2.2 Investigations

Initial laboratory investigations given in Table 1 demonstrated leukocytosis, deranged liver function, acute kidney injury, electrolyte disturbances with marked hypokalemia, coagulopathy, and metabolic alkalosis. These findings were consistent with sepsis secondary to urinary tract infection, complicated by multi‐organ involvement. The patient was managed with broad‐spectrum intravenous antibiotics, fresh frozen plasma, vitamin K, dual inotropic support, and central venous pressure guided fluid and potassium replacement. Over the subsequent days, serial monitoring given in Table 2 showed improvement in infection and renal function parameters, with normalization of blood counts and liver enzymes. However, despite appropriate potassium supplementation, hypokalemia persisted along with metabolic alkalosis. This raised the suspicion of an underlying renal potassium‐wasting disorder. Targeted investigations given in Table 3 revealed normal magnesium levels, increased urinary sodium, potassium, and chloride losses, and a normal to high urine calcium to creatinine ratio. Together with the presence of metabolic alkalosis, these findings helped exclude Gitelman syndrome and favored the diagnosis of Bartter syndrome. A detailed and repeated medication history was obtained, specifically screening for loop and thiazide diuretics, laxative abuse, and nephrotoxic agents including aminoglycosides, amphotericin B, and cisplatin. No history of exposure to such agents was identified. There was no clinical evidence of chronic gastrointestinal losses, surreptitious diuretic intake, or substance misuse. Collateral history was obtained where appropriate to further substantiate the absence of exogenous contributors.

**TABLE 1:** Baseline laboratory investigations on admission.

Investigations	Lab values
WBC	35,000/microliter
Hb	7.8 g/dL
Hct	35%
MCV	81
Platelet	151,000/μL
Neutrophil	79%
Lymphocytes	20%
ESR	32
LDH	1008
AST	481
ALT	442
Bilirubin	3.7
Alkaline phosphatase	111
Creatinine	4 mg/dL
Urea	111 mg/dL
APTT	59 s
PT	27
Sodium	130 mmol/L
Potassium	2.5 mmol/L
Chloride	83 mmol/L
ABGs	
Ph	7.45
PCO2	35 mHg
PaO2	65 mmHg
HCO3	27 mmol
CRP	55 mg/L
Troponin I	Negative
D‐Dimers	100
FDPs	< 1

**TABLE 2:** Serial hematological, biochemical, and electrolyte trends during hospital stay.

Investigations	Day 1	Day 2	Day 3
WBC	15,000/microliter	13,000/microliter	10,000/microlite
Hb	8.og/dL	8.3 g/dL	8.4 g/dL
Hct	40%	43%	42%
MCV	80	83	84
Platelet	251,000/μL	252,000/μL	257,000/μL
Neutrophil %	80%	78%	77%
Lymphocytes %	15%	16%	17%
ESR	10	11	10
AST	312	112	87
ALT	345	137	78
Bilirubin	1.3	1.1	1.0
Alkaline phosphatase	311	270	260
Serum electrolytes	Day 1	Day 2	Day 3
Sodium	132 mmol/L	133 mmol/L	136 mmol/L
Potassium	2.3 mmol/l (Replacement given)	2.5 mmol/l (Replacement given)	2.4 mmol/L (Replacement given)
Chloride	87 mmol/L	81 mmol/L	85 mmol/L
Renal function test	Day 1	Day 2	Day 3
Creatinine	1.3 mg/dL	1.1 mg/dl	1.2 mg/dL
Urea	30 mg/dL	24 mg/dL	20 mg/dL
Urine R/E	Shows 4–5 Pus cells Urine PH 5.1	Shows 2–3 Pus cells Urine PH 5.1	Shows 2–3 Pus cells Urine PH 5.0
ABG	Metabolic alkalosis	Metabolic alkalosis	Metabolic alkalosis

**TABLE 3:** Specialized investigations for differential diagnosis of hypokalemia.

Invetigations	Lab values
Serum Magnesium	1.9 mg/dL (1.7 to 2.2)
Urine electrolytes	
Sodium	30 mEq/L (> 20 mEq/L)
Potassium	38 mEq/L (15–20 meq/L)
Chloride	52 mmol/L (20–40 mmol/L)
ABG	Showed Metabolic Alkalosis
Spot urine calcium/creatinine ratio	150 mg/g (200 mg/g or 565 mmol/mol)

The chronicity of symptoms, stable renal function, and biochemical profile demonstrating persistent hypokalemic metabolic alkalosis consistent with renal salt wasting supported a primary tubular disorder rather than an acquired etiology. We have expanded the relevant section of the manuscript to clarify this differential exclusion.

### 2.3 Treatment

Following nephrology consultation, a final diagnosis of Bartter syndrome was established. The patient was commenced on oral potassium chloride supplementation and spironolactone.

### 2.4 Outcomes and Follow‐Up

At the time of discharge, lab investigation given in Table 4 revealed his renal function had normalized, serum potassium improved, and metabolic alkalosis resolved. He was discharged in stable condition with advice for weekly nephrology follow‐up and regular monitoring of renal function and electrolytes. On follow‐up, he showed gradual clinical and biochemical improvement with oral therapy.

**TABLE 4:** Laboratory values post treatment on day of discharge.

Investigations	Lab values
Creatinine	1.1 mg/dL
Urea	25 mg/dL
Serum electrolytes	
Sodium	138 mmol/L
Potassium	3.5 mmol/l
Chloride	87 mmol/l
Urine R/E	Urine PH 5.1
ABG	Showed PH 7.43 PCO2 38 HCO3 26

## 3 Discussion

Our patient presented with persistent hypokalemia despite adequate replacement therapy, normal blood pressure, and metabolic alkalosis, which raised suspicion of a renal salt‐wasting disorder. Similar to our findings, previous case reports have highlighted that Bartter syndrome often presents with refractory hypokalemia and normotension, and that the combination of hypokalemia with metabolic alkalosis in the absence of hypertension is highly suggestive of a renal salt‐wasting disorder [6].

In this case, the presence of renal potassium wasting with normal magnesium levels and a raised urinary calcium‐to‐creatinine ratio helped differentiate Bartter syndrome from Gitelman syndrome. This diagnostic approach aligns with the established literature, where hypocalciuria is typically associated with Gitelman syndrome, whereas hypercalciuria or normocalciuria points toward Bartter syndrome [7]. Therefore, the patient's biochemical profile strongly supported a diagnosis of Bartter syndrome.

Another notable feature was that our patient had a history of myocardial infarction and mild systolic dysfunction with persistent hypotension, a presentation not commonly reported in adults with Bartter syndrome. While Bartter syndrome primarily affects renal tubular function, chronic hypokalemia can contribute to arrhythmias and myocardial instability, making cardiac involvement an important clinical consideration [2].

Furthermore, despite appropriate potassium supplementation, the hypokalemia and metabolic alkalosis persisted until potassium‐sparing diuretics were introduced, which resulted in marked improvement. This treatment response is consistent with the literature, where combined therapy with potassium supplements, spironolactone, and nonsteroidal anti‐inflammatory drugs (NSAIDs) has shown effective correction of electrolyte imbalance and symptom improvement [8].

Bartter syndrome remains a rare but important differential diagnosis in adults presenting with refractory hypokalemia, particularly when other common causes such as diuretic abuse, vomiting, or renal tubular acidosis have been excluded [9]. As reported in prior reviews, late‐diagnosed Bartter syndrome cases often go undiagnosed until adulthood because of overlapping features with more prevalent conditions [3].

Our patient showed gradual clinical and biochemical improvement with oral therapy on follow‐up, emphasizing the importance of long‐term monitoring. Long‐term outcomes depend on early recognition and strict follow up, as untreated hypokalemia may lead to progressive renal impairment and cardiovascular complications [10].

In conclusion, this case reinforces the importance of considering Bartter syndrome in adults presenting with persistent hypokalemia and metabolic alkalosis, particularly when unresponsive to conventional potassium supplementation. The diagnosis should be suspected in the presence of normal blood pressure, elevated urinary potassium and chloride losses, and exclusion of secondary causes. Early recognition and prompt initiation of potassium‐sparing agents, along with regular monitoring of renal and cardiac function, are essential to prevent long term complications. Awareness of such atypical adult presentations can aid clinicians in timely diagnosis and management of this rare but treatable tubulopathy.

Genetic testing was not performed due to practical and systemic limitations. Molecular diagnostics for Bartter syndrome are not available at our institution, and access to specialized genetic facilities in our region is limited. Furthermore, the financial cost of testing posed a significant burden, and despite detailed counseling regarding its diagnostic value, the patient declined further evaluation.

In this context, the diagnosis was established based on a comprehensive clinical assessment supported by characteristic biochemical findings consistent with a primary renal tubular salt‐wasting disorder. While genetic confirmation would have enhanced diagnostic precision, well‐defined clinical and laboratory criteria remain fundamental to diagnosis, particularly in resource‐constrained settings.

## 4 Conclusion

Bartter syndrome, though classically a pediatric renal tubulopathy, can occasionally present in adulthood with non‐specific symptoms such as weakness, hypotension, and refractory hypokalemia. This case highlights the diagnostic value of integrating clinical suspicion with targeted biochemical investigations to differentiate Bartter syndrome from other causes of metabolic alkalosis, particularly Gitelman syndrome. Persistent hypokalemia despite adequate replacement therapy should prompt evaluation for underlying tubular defects. Early diagnosis and appropriate management with potassium supplementation and potassium‐sparing diuretics lead to favorable outcomes and help prevent renal and cardiovascular complications. Greater clinical awareness of this rare entity is essential to avoid diagnostic delays, especially in resource‐limited settings where genetic testing may not be readily available.

## Author Contributions

**Muhammad Asif:** conceptualization, supervision. **Ayesha Sanam:** data curation, investigation, visualization. **Muhammad Ilyas:** methodology, writing – original draft. **Sawera Tahir:** methodology, validation, visualization, writing – original draft. **Tahir Hanif:** methodology, writing – original draft. **Muddassir Khalid:** project administration, supervision, writing – review and editing. **Iqra Sarwar:** methodology, writing – review and editing. **Fred Segawa:** conceptualization, writing – review and editing.

## Funding

The authors have nothing to report.

## Disclosure

Peer and Provenance Statement: Not commissioned, externally peer‐reviewed.

## Ethics Statement

The authors have nothing to report.

## Consent

Written informed consent was obtained from the patient for publication of this case report and accompanying images. A copy of the written consent is available for review by the Editor‐in‐Chief of this journal on request.

## Conflicts of Interest

The authors declare no conflicts of interest.

## Data Availability

Data available on request from the authors.
